# Conditional Inducible Triple-Transgenic Mouse Model for Rapid Real-Time Detection of HCV NS3/4A Protease Activity

**DOI:** 10.1371/journal.pone.0150894

**Published:** 2016-03-04

**Authors:** Min Yao, Xin Lu, Yingfeng Lei, Jing Yang, Haiwei Zhao, Qinghua Qiao, Peijun Han, Zhikai Xu, Wen Yin

**Affiliations:** 1 Department of Microbiology, Fourth Military Medical University, Xi’an, China; 2 Department of Blood Transfusion, Xijng Hospital, Fourth Military Medical University, Xi’an, China; University of Padua, ITALY

## Abstract

Hepatitis C virus (HCV) frequently establishes persistent infections that can develop into severe liver disease. The HCV NS3/4A serine protease is not only essential for viral replication but also cleaves multiple cellular targets that block downstream interferon activation. Therefore, NS3/4A is an ideal target for the development of anti-HCV drugs and inhibitors. In the current study, we generated a novel *NS3/4A/Lap/LC-1* triple-transgenic mouse model that can be used to evaluate and screen NS3/4A protease inhibitors. The NS3/4A protease could be conditionally inducibly expressed in the livers of the triple-transgenic mice using a dual Tet-On and Cre/loxP system. In this system, doxycycline (Dox) induction resulted in the secretion of *Gaussia* luciferase (Gluc) into the blood, and this secretion was dependent on NS3/4A protease-mediated cleavage at the *4B5A* junction. Accordingly, NS3/4A protease activity could be quickly assessed in real time simply by monitoring Gluc activity in plasma. The results from such monitoring showed a 70-fold increase in Gluc activity levels in plasma samples collected from the triple-transgenic mice after Dox induction. Additionally, this enhanced plasma Gluc activity was well correlated with the induction of NS3/4A protease expression in the liver. Following oral administration of the commercial NS3/4A-specific inhibitors telaprevir and boceprevir, plasma Gluc activity was reduced by 50% and 65%, respectively. Overall, our novel transgenic mouse model offers a rapid real-time method to evaluate and screen potential NS3/4A protease inhibitors.

## Introduction

At least 150 million people are chronically infected with hepatitis C virus (HCV) worldwide. The majority of these individuals are at significant risk of developing severe liver diseases, including liver fibrosis, liver cirrhosis, and hepatocellular carcinoma [1,2]. A member of the *Flaviviridae* family, HCV is an enveloped virus that contains a positive-strand, 9.6-kb-long RNA genome [3]. Translation of the HCV genome results in the formation of a polyprotein that is subsequently cleaved into three structural proteins (core, E1 and E2) and seven nonstructural (NS) proteins (p7, NS2, NS3, NS4A, NS4B, NS5A and NS5B) through the actions of two host proteases and two viral proteases (NS2 and NS3) [4]. The N-terminal portion of NS4A is responsible for the membrane association of the NS3/4A complex [5]. Using NS4A as a co-factor, the NS3/4A serine protease is not only vital for viral replication [3] but also cleaves multiple cellular targets that block downstream interferon activation [3,6]. Thus, NS3/4A is a promising target for the development of anti-HCV drugs. Currently, no small animal model is available to test NS3/4A inhibitors *in vivo* that provides rapid, real time, and reproducible results.

HCV only readily infects humans and chimpanzees, which makes studying HCV infection and testing novel therapeutics *in vivo* challenging [7]. A multitude of approaches for modeling HCV infection *in vivo* have been examined, including the creation of transgenic mice that express either individual or combinations of HCV proteins or essential HCV host factors, the assessment of mice transplanted with human hepatocytes, and the evaluation of small non-human primates [8,9]. The use of such animal models has been beneficial for the study of HCV infection and in testing novel HCV inhibitors, but additional models are still needed.

In the current study, we report the creation of a novel *NS3/4A/Lap/LC-1* triple-transgenic mouse model. In this model, the NS3/4A protease can be conditionally expressed in the liver following induction with doxycycline (Dox). Using this set-up, NS3/4A protease activity can be rapidly assessed in real time simply by monitoring *Gaussia* luciferase (Gluc) activity in plasma. This novel transgenic mouse model offers an attractive platform for evaluating and screening NS3/4A protease inhibitors.

## Materials and Methods

### Plasmid construction

The recombinant *pBI-NS3/4A* vector was constructed based on the previously constructed *pBI-G//LoxP-Fluc-BGH PolyA-LoxP-NS3/4A* plasmid [10]. The entire *TM-4B5A-Gluc* gene (716 bp) was synthetized by GenScript Biotech (Nanjing, China) and cloned into the *pUC57* vector (Clontech, Mountain View, CA, USA) to generate the *pUC57-TM-4B5A-Gluc* plasmid. Next, the *TM-4B5A-Gluc* cassette was excised as an *Xba I*-blunted fragment and transferred into the blunted *Xba I* site of the *pBI-G//LoxP-Fluc-BGH PolyA-LoxP-NS3/4A* plasmid to generate the final *pBI-NS3/4A* transgene.

*pTet-On-rtTA* expresses a reverse tetracycline-controlled transactivator (rtTA) under the *Lap* promoter, which can only bind to the tetracycline operator (TetO) and activate transcription in the presence of Dox. In addition, *pBI-Cre* was also used to enable the conditional expression of Cre recombinase under TetO, which deletes genes flanked by the *loxP* sequence [11].

### Verification of the functionality of the *pBI-NS3/4A* transgene *in vitro*

Chinese hamster ovary (CHO) cells were cultivated in RPMI-1640 media supplemented with 10% fetal bovine serum (Life Technologies, USA). The functionality of *pBI-NS3/4A* was tested through co-transfection along with the *pTet-On* and *pBI-G//Cre* plasmids (Clontech, Mountain View, CA, USA). To accomplish this, CHO cells were transfected using Lipofectamine 2000 reagent (Life Technologies, Grand Island, NY, USA) according to the manufacturer’s protocol. After co-transfection for 6 h, the culture medium was replaced with fresh medium containing 1 μg/mL Dox (Sigma-Aldrich, St. Louis, MO, USA), and induction proceeded for 48 h. Subsequently, the cell medium and cells were separately collected to assay luciferase activity via bioluminescent imaging (BLI) and to assay luciferase expression via western blot analysis.

### Generation and screening of an *NS3/4A* transgenic founder mouse

This study was conducted under strict adherence to the Guide for the Care and Use of Laboratory Animals published by the National Institutes of Health. The study protocol was approved by the Committee on the Ethics of Animal Experiments of the Fourth Military Medical University (Permit Number: 20100530–20). All efforts were made to minimize animal suffering.

The *pBI-NS3/4A* plasmid was linearized using *Ase I* (New England Biolabs, Beverly, MA, USA). *NS3/4A* transgenic founder mice were obtained through pronuclear injection of the purified *pBI-NS3/4A* fragment into fertilized eggs. First, the *NS3/4A* transgenic founder mice were crossed with age-matched wild-type C57BL/6 mice (WT mice). Subsequently, homozygous *NS3/4A* transgenic mice were generated after repeatedly crossing siblings. All mice were housed under specific-pathogen-free (SPF) conditions at the Animal Center of the Fourth Military Medical University. For PCR-based genotyping, F_ns3/4a_ and R_ns3/4a_ primers (F_ns3/4a_: 5'-ACT TGA AGG GCT CTT CGG-3' and R_ns3/4a_: 5'-TTG GTT ATG GGG TGT GTG-3') were used with Premix Taq Version 2.0 (Takara, Dalian, China) according to the manufacturer’s protocol.

### Screening of *NS3/4A/Lap/LC-1* triple-transgenic mice

Both the *Lap* and *LC-1* transgenic lines were kind gifts from Hermann Bujard (University of Heidelberg, Germany) [12,13]. *Lap/LC-1* dual transgenic mice were generated after crossing *Lap* transgenic and *LC-1* transgenic mice [10]. Following this, 6- to 8-week-old homozygous *NS3/4A* mice (*ns3/4a*^*+/+*^) were crossed with age-matched *Lap/LC-1* mice, and the offspring were genotyped by PCR. BLI was used to detect firefly luciferase (Fluc) and Gluc activity to obtain phenotype-positive *NS3/4A/Lap/LC-1* triple-transgenic mice.

Genotyping PCR was performed using the F_ns3/4a_ and R_ns3/4a_, F_rtTA_ and R_rtTA_ and F_Cre_ and R_Cre_ primer pairs (F_rtTA_: 5'-CCA TGT CTA GAC TGG ACA AGA-3'; R_rtTA_: 5'-ctc cag gcc aca tat gat tag-3'; F_Cre_: 5'-GTA ATC TGG CAT TTC TGG G-3'; R_Cre_: 5'-CAC CAG CTT GCA TGA TCT C-3'). Following this, genotyping-positive triple-transgenic mice were induced with Dox (1 mg/mL Dox and 50 g/L sugar were dissolved in their drinking water) for 3 days and further screened via BLI to detect Fluc signals. For verification purposes, we collected plasma and urine from the Fluc-positive mice (Fluc^+^ mice) to detect Gluc activity after 3 days of Dox induction. The mice were then sacrificed to obtain liver samples for western blot analysis, histology and immunohistochemistry.

### Assessment of luciferase activity *in vitro*

Fluc and Gluc activity assays were performed using Firefly Luciferase and Gaussia Luciferase Assay Kits (Thermo Scientific Pierce, Rockford, IL, USA) according to the manufacturer’s instructions. Luciferase activity was measured over 10 sec using a luminometer (Promega GloMax 20/20). Additionally, total protein content in cell lysates was determined using BCA reagent (Thermo Scientific Pierce, Rockford, IL, USA) according to the manufacturer’s instructions, and the data were expressed as relative light units (RLU)/mg total protein or RLU/μL plasma. All experiments were performed in triplicate and repeated at least twice.

### Bioluminescent imaging of luciferase expression

For BLI of Fluc activity in co-transfected cells, the culture medium was removed, and 100 μL (1 mg/mL in PBS) D-luciferin was added to each well. For BLI of Gluc activity, 100 μL coelenterazine-SOL (100 μM, NanoLight Technology, USA) was added to the culture medium and the cells and the culture medium were measured over 10 sec using an IVIS Imaging System (Caliper Life Sciences, Hopkinton, MA, USA).

For *in vivo* BLI, after 3 days of Dox induction, mice were intraperitoneally injected with D-luciferin (150 mg/kg body weight; Caliper Life Sciences, Hopkinton, MA, USA) or INJ Inject-A-Lume Coelenterazine (100 μg per 25-g mouse, NanoLight Technology, USA). The treated mice were anesthetized with isoflurane (Baxter, Cambridge, MA, USA), and RLU were measured over 10 sec using an IVIS Imaging System. Regions of interest (ROI) were automatically drawn. The bioluminescence signal was represented as photons/s/cm^2^/sr.

### Western blotting

A 40-μg aliquot of cell lysate or liver homogenate was electrophoresed on a 12% polyacrylamide gel and transferred to a polyvinylidene difluoride (PVDF) membrane (Millipore, Billerica, MA, USA). Western blotting was performed according to a standard protocol. Protein detection was performed using a rabbit polyclonal antibody against Gluc (1:1000, Abcam, Cambridge, MA, USA), a mouse monoclonal antibody against NS3 protease [10] (1:200), and a mouse β-actin antibody (Sigma, St. Louis, MO, USA). Goat anti-rabbit secondary antibodies and goat anti-mouse secondary antibodies labeled with IRDye infrared dyes (LI-COR Biosciences, Lincoln, NE, USA) were used for infrared fluorescence detection. Fluorescent signals on the PVDF membrane were visualized using an Odyssey Infrared Imaging System (LI-COR Biosciences, Lincoln, NE, USA).

### Histology, immunohistochemistry and serum biochemistry

Liver tissues were fixed in 10% neutral buffered formalin for at least 24 h and subsequently embedded in paraffin. Tissue sections (5 μm) were affixed to slides, deparaffinized and stained with hematoxylin & eosin (H&E) to determine morphological changes. For immunohistochemical staining, antigen retrieval was performed using citric acid buffer. The tissue sections were incubated with a primary antibody against NS3 (1:100, orb10789, Biorbyt) overnight. Next, a GTVision^™^ III detection system/Mo&Rb was applied (GK500711, Taiwan). Images were acquired using an Olympus BX60 microscope (Japan). Serum biochemistry was evaluated using a TBA-200FR automated clinical chemistry analyzer (Toshiba, Tokyo, Japan).

### Using triple-transgenic mice to evaluate the effects of NS3/4A inhibitors

Telaprevir and boceprevir were purchased from MedChem Express (Pumai Biotechnology Co., LTD, Shanghai, China). To evaluate the effect of telaprevir, triple-transgenic mice were randomized into two groups (n = 5 per group) and administered either telaprevir (200 mg/kg) or vehicle (dimethyl sulfoxide, DMSO) via oral gavage twice daily for 10 days. At the same time, the mice were continuously induced with Dox (1 mg/mL Dox and 50 g/L sugar were dissolved in their drinking water).

To evaluate the effect of boceprevir, triple-transgenic mice were induced with Dox for 10 days (n = 5 per group). On the third day after Dox induction, when plasma Gluc activity reached its peak, the mice were administered either boceprevir (100 mg/kg) or DMSO via oral gavage twice daily for 7 days. During this period, blood was collected from the caudal vein daily to detect plasma Gluc activity.

### Statistical analyses

All data are expressed as the means ± standard deviations (SDs). The statistical significance of the obtained data was analyzed using Student’s t-test in the GraphPad Prism 5 software package (GraphPad Software, San Diego, CA). Differences with p-values < 0.05 were considered statistically significant.

## Results

### Correlation between HCV NS3/4A protease and Gluc activities *in vitro*

**Fig 1:**
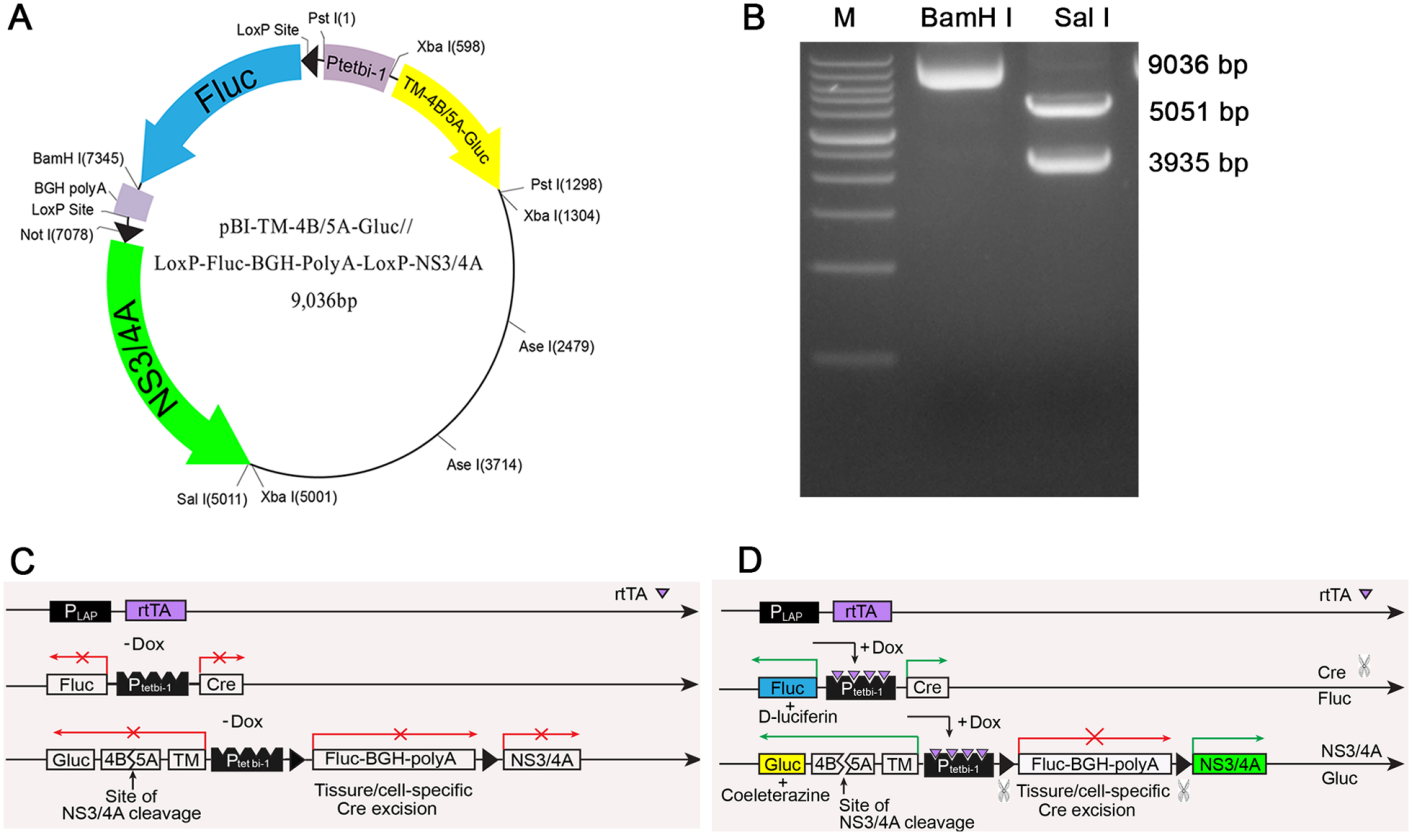
Schematic overview of the created conditional inducible triple-transgenic mouse model for rapid real-time detection of HCV NS3/4A protease activity. **(A)** The *pBI-NS3/4A* transgenic vector. **(B)** The *pBI-NS3/4A* vector was digested with *Bam HI* and *Sal I* and submitted to gel electrophoresis to confirm the correct construction. M: 500–12,000 bp. **(C)** Construction of the *pTet-On-rtTA*, *pBI-Cre* and *pBI-NS3/4A* plasmids. rtTA is expressed under the *Lap* promoter, but without Dox induction, rtTA is unable to bind to P_tet_bi-1 to provide temporal control of gene expression. **(D)** Schematic overview of the created triple-transgenic mouse model for rapid real-time assessment of HCV NS3/4A protease activity. In the presence of Dox, rtTA binds to the P_tet_bi-1 sequence on the *pBI-Cre* and *pBI-NS3/4A* vectors and activates transcription of the *Cre* and *Fluc* genes in *pBI-Cre*. Cre-mediated removal of the *Fluc-BGH-PolyA* STOP cassette leads to *NS3/4A* expression from the *pBI-NS3/4A* plasmid. The NS3/4A protease then cleaves at the *4B5A* junction, resulting in the secretion of Gluc into either culture medium or blood. TM, C-terminal transmembrane domain; BGH, bovine growth hormone.

The *pBI-NS3/4A* vector harbors Gluc and Fluc reporter genes under a bidirectional tet-responsive promoter. On one side of the promoter, the C-terminal transmembrane domain (*TM*) was designed to direct Gluc to the outer mitochondrial membrane, thereby preventing Gluc secretion [14,15]. In addition, *4B5A*, the NS3/4A protease recognition cleavage sequence [3,6], was inserted between the *TM* sequence and the Gluc reporter gene. On the other side of the promoter, a *loxP*-flanked *Fluc-BGH-polyA* transcriptional STOP cassette prevented the expression of *NS3/4A* mRNA (Fig 1A). The *pBI-NS3/4A* vector was digested with *Bam HI* and *Sal I* and sequenced to confirm the correct construction (Fig 1B).

Without Dox induction, the above vector did not express *NS3/4A* (Fig 1C). However, after the administration of Dox, the rtTA in the vector undergoes nuclear translocation and activates P_tet_bi-1, which induces the expression of Cre and the Fluc reporter gene. Cre recombination leads to the specific deletion of the STOP cassette, resulting in the expression of the NS3/4A protease and consequent cleavage at the *4B5A* junction. This cleavage results in the secretion of Gluc into either cell culture medium or blood (Fig 1D). Gluc activity can then be monitored to provide rapid real-time assessment of NS3/4A protease expression *in vitro* or *in vivo*.

**Fig 2:**
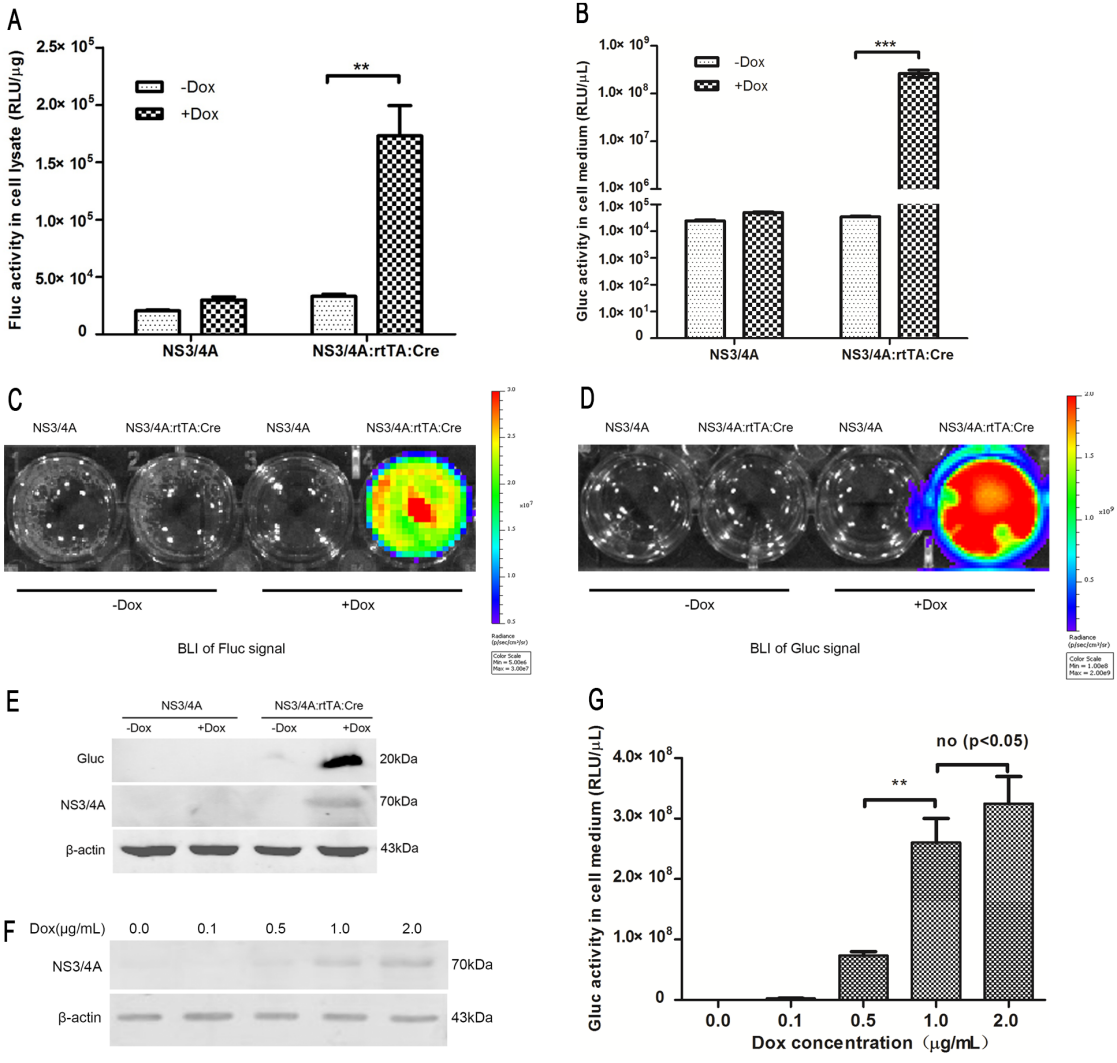
Verification of the functionality of the *pBI-NS3/4A* plasmid *in vitro*. **(A)** The *pBI-NS3/4A* plasmid was co-transfected into CHO cells with *pTet-On-rtTA* and *pBI-Cre* or *pBI-NS3/4A* alone as a control. The cells were treated with Dox (l μg/mL) at 6 h after transfection and harvested 48 h after transfection. Cell lysates were measured for Fluc activity. **(B)** Gluc activity was measured in the culture medium. **(C)** BLI was used to assess Fluc signals in the co-transfected cells. To accomplish this, 100 μL D-luciferin (1 mg/mL in PBS) was added to the cells, and they were then scanned using an IVIS Imaging System. **(D)** BLI was used to assess Gluc signals in the culture medium of the co-transfected cells. To accomplish this, 100 μL coelenterazine-SOL (100 μM) was added to the culture medium, and the mixture was then scanned using an IVIS Imaging System. **(E)** A representative western blot. Samples of culture medium from the co-transfected cells were concentrated and subsequently analyzed using a rabbit anti-Gluc antibody. Cell lysates from the co-transfected cells were analyzed using a mouse anti-NS3/4A antibody. **(F)** The co-transfected cells were induced with various concentrations of Dox (0.l, 0.5, l, and 2 μg/mL). NS3/4A protease expression in the co-transfected cells was analyzed via western blotting. **(G)** Samples of the culture medium from the co-transfected cells were assessed using a Gluc activity assay.

To verify the functionality of the *pBI-NS3/4A* transgene, the *pBI-NS3/4A* plasmid was co-transfected with the *pTet-On-rtTA* and *pBI-Cre* plasmids into CHO cells. The co-transfected cells were treated with Dox to induce gene expression and subsequently evaluated for intracellular Fluc activity and extracellular Gluc activity (both measured as RLU) in culture medium via luciferase activity assays and BLI. The Fluc activity assay revealed significantly greater Fluc activity in the cell lysates of the co-transfected cells that were induced with Dox compared to the non-induced co-transfected and *pBI-NS3/4A*-transfected cells at 48 h post-transfection (Fig 2A). Similarly, the Gluc activity in the culture medium of the co-transfected cells that were induced with Dox was approximately 1000-fold higher than that of the non-induced co-transfected cells (Fig 2B). BLI also revealed strong Fluc activity in the co-transfected cells and strong Gluc activity in the culture medium after Dox induction (Fig 2C and 2D). No Gluc or Fluc activity was observed in the *pBI-NS3/4A*-transfected cells or the non-induced co-transfected cells. Western blotting confirmed that the NS3/4A protease was expressed in the co-transfected cells. Additionally, we confirmed that Gluc was only secreted into the culture medium of the co-transfected cells following Dox induction (Fig 2E).

To further confirm that the increased Gluc activity observed in the culture medium of the co-transfected cells was associated with the expression of the NS3/4A protease, we used serial dilutions of Dox to induce the cells. Western blotting analysis showed that NS3/4A expression gradually increased as the Dox concentration increased from 0.1 to 2 μg/mL (Fig 2F). In addition, Gluc activity in the culture medium increased as the concentration of Dox increased (Fig 2G). These results demonstrated that the NS3/4A protease could be conditionally inducibly expressed under the control of the Tet-On and Cre/loxP systems. They also confirmed that Gluc activity in the culture medium paralleled intracellular NS3/4A protease expression. Collectively, the above results provide a foundation for establishing a conditional inducible mouse model that can offer rapid real-time detection of HCV NS3/4A protease activity.

### Generation and characterization of *NS3/4A/Lap/LC-1* transgenic mice

**Fig 3:**
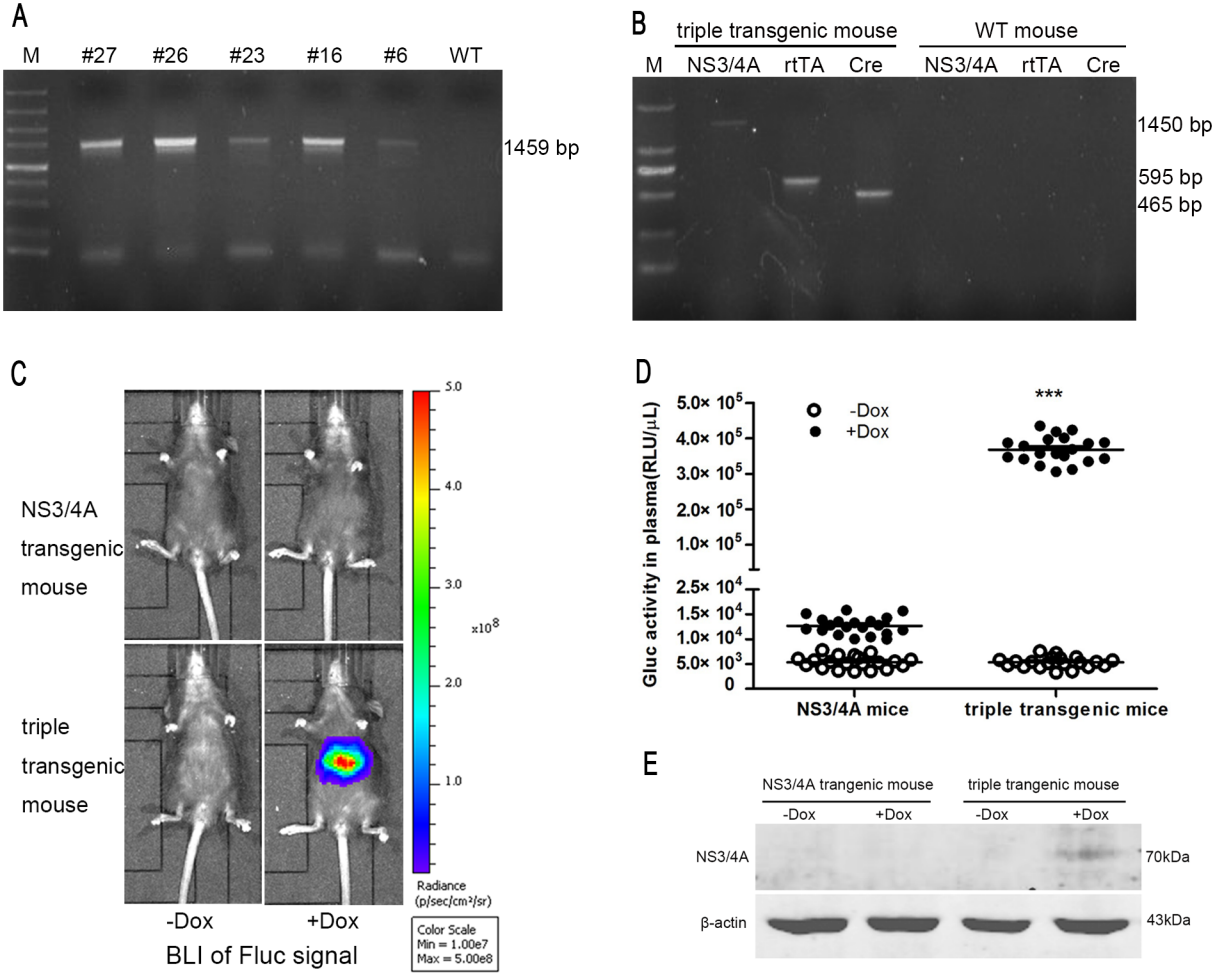
Generation and verification of the functionality of *NS3/4A/Lap/LC-1* triple-transgenic mice. **(A)** PCR analysis of *NS3/4A* transgenic mice. M: DL 500–12,000; WT: wild type. **(B)** PCR analysis of triple-transgenic mice that contained the *NS3/4A*, *rtTA* and *Cre* transgenes. The results are representative of a single triple-transgenic mouse. M: DL 5000. **(C)** Representative BLI of Fluc signal. *NS3/4A* transgenic mice and triple-transgenic mice were induced with Dox (1 mg/mL Dox and 50 g/L sugar were dissolved in their drinking water) for 3 days. Then, the mice were intraperitoneally injected with D-luciferin and scanned using an IVIS imaging system. Non-Dox-induced *NS3/4A* transgenic mice and triple-transgenic mice were used as negative controls. **(D)** Gluc activity (shown in RLU) in the plasma of *NS3/4A* transgenic mice and triple-transgenic mice prior to and after Dox induction for 3 days (n = 20 mice/group). Each symbol represents an individual animal. The horizontal bars indicate group medians. *** = p<0.001 compared to the negative control. **(E)** Representative western blot analysis. Liver homogenates from *NS3/4A* transgenic mice and triple-transgenic mice induced with Dox for 3 days were analyzed via western blotting using anti-NS3 and anti-β-actin antibodies.

Five *NS3/4A* transgenic founder mice (F_0_#27, F_0_#26, F_0_#23, F_0_#16 and F_0_#6) were produced by pronuclear injection. Each founder mouse was verified to contain the *NS3/4A* (1450 bp) fragment based on PCR analysis using a primer set specific for *NS3/4A* (Fig 3A). Because mouse F_0_ #26 had a high copy number of the transgene and was the most fertile, this mouse was used to establish transgenic lines. The *NS3/4A* transgenic mice were passaged to the sixth generation (F_6_) after crossing siblings to obtain homozygous transgenic mice. The homozygous *NS3/4A* transgenic mice were healthy and fertile and used to maintain the line in a homozygous state.

Next, *NS3/4A/Lap/LC-1* triple-transgenic mice were generated by mating age-matched *NS3/4A* transgenic mice with *Lap/LC-1* dual transgenic mice that specifically and conditionally expressed Cre recombinase in the liver under the control of the Tet-On system. Genotyping PCR indicated that the triple-transgenic mice simultaneously contained the *NS3/4A* (1450 bp), *rtTA* (595 bp) and *Cre* transgenes (465 bp) (Fig 3B). BLI showed that approximately 70% of the genotyping-positive mice exhibited intense Fluc signals specifically in the liver area after Dox induction for 3 days (Fig 3C).

**Fig 4:**
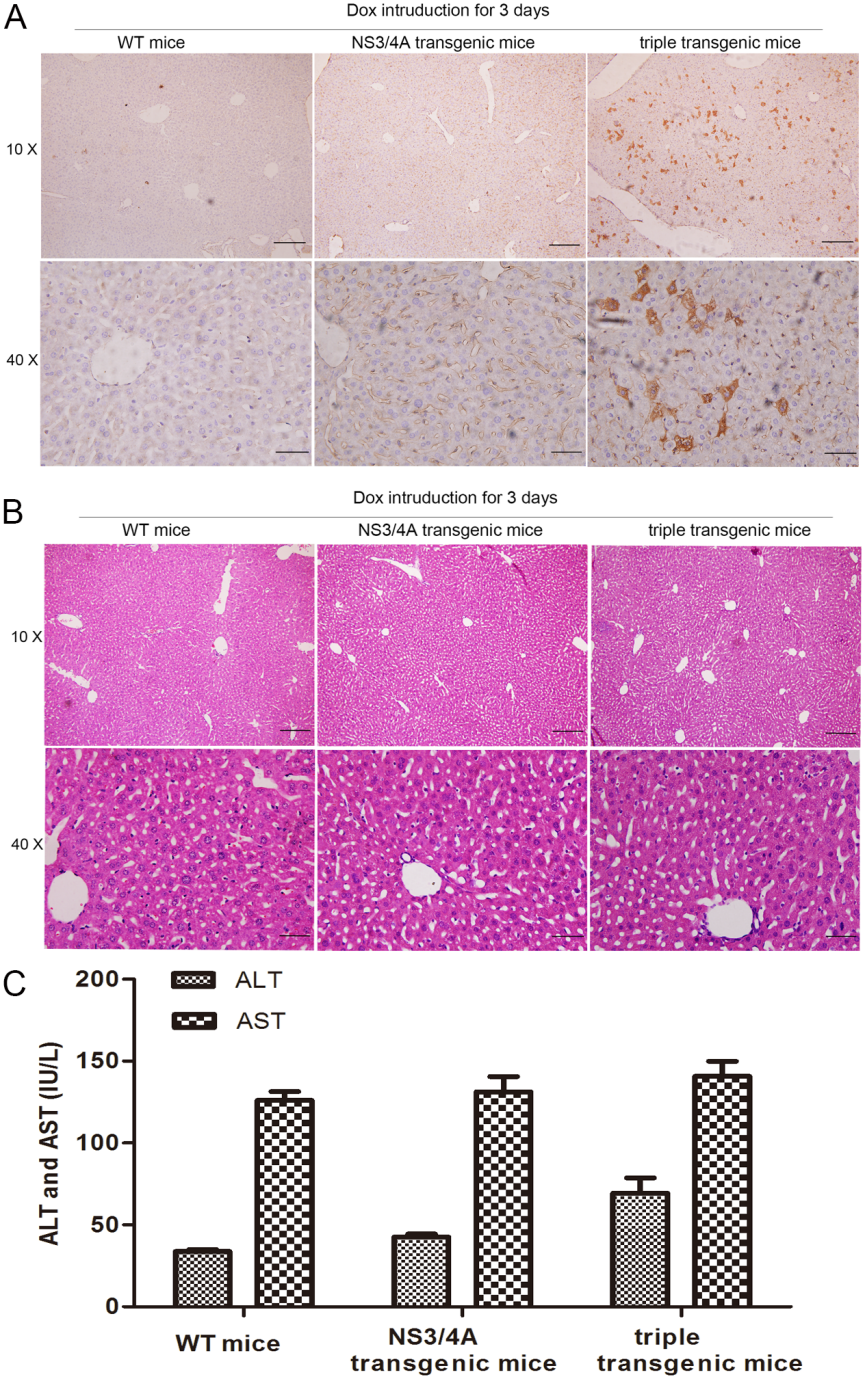
Liver damage caused by NS3/4A protease expression in the triple-transgenic mice. **(A)** Representative image of immunohistochemical staining of the NS3/4A protease in liver tissue sections from a representative WT mouse (left column), *NS3/4A* transgenic mouse (middle column) and triple-transgenic mouse (right column). The mice were induced with Dox for 3 days (1 mg/mL Dox and 50 g/L sugar were dissolved in their drinking water). The slides were counterstained with H&E. The upper images were taken at 10X magnification (scale bars, 200 μm). The lower images were taken at 40 X magnification (scale bars, 50 μm). **(B)** Histological examination of tissue damage. Liver sections from a representative WT mouse (left column), *NS3/4A* transgenic mouse (middle column) and triple-transgenic mouse (right column) following induction with Dox for 3 days. The sections were stained with H&E. The upper images were taken at 10 X magnification (scale bars, 200 μm). The lower images were taken at 40 X magnification (scale bars, 50 μm). **(C)** Analysis of serum ALT and AST levels in WT mice, *NS3/4A* transgenic mice and triple-transgenic mice following induction with Dox for 3 days (n = 10).

For verification purposes, plasma and urine samples were collected from the Fluc^+^ triple-transgenic mice after induction with Dox for 3 days to assess Gluc activity. These mice exhibited approximately 70-fold greater Gluc activity than that observed prior to induction (pre-treatment: 5.33×10^3^±245.5 RLU/μL plasma, post-treatment: 3.68×10^5^±8232 RLU/μL plasma, n = 20) (Fig 3D). After Dox induction, plasma Gluc RLU values also increased slightly in the *NS3/4A* transgenic mice (pre-treatment: 5.37×10^3^±283.1 RLU/μL plasma, post-treatment: 1.27×10^4^±392.8 RLU/μL plasma, n = 20) (Fig 3D). However, the Dox-induced triple-transgenic mice exhibited approximately 30-fold higher plasma Gluc RLU values compared to the Dox-induced *NS3/4A* transgenic mice. Western blotting analysis of liver homogenates indicated that the NS3/4A protease was specifically expressed in the Dox-induced triple-transgenic mice (Fig 3E). Additionally, immunohistochemical staining showed that approximately 10% of the hepatocytes in these mice expressed the NS3/4A protease, whereas *NS3/4A* expression was not detected in the Dox-induced *NS3/4A* transgenic mice or the Dox-induced WT mice. We also observed that hepatic sinusoidal endothelial cells were stained darker in the Dox-induced *NS3/4A* transgenic mice and the triple-transgenic mice compared to WT mice (Fig 4A).

Collectively, the above results demonstrated that the expression of the *NS3/4A* transgene in the triple-transgenic mice was conditional and could be induced under the control of the dual Tet-On and Cre/loxP system. The results also suggested that enhanced Gluc activity in the plasma was associated with increased NS3/4A protease expression in the liver. Thus, the results of our *in vivo* and *in vitro* assays were completely consistent, and we concluded that a triple-transgenic mouse model possessing inducible conditional expression of the HCV NS3/4A protease was successfully created. In this mouse model, plasma Gluc activity can be used as a rapid real-time reporter of NS3/4A activity. As such, this novel model was next used to evaluate the effects of different NS3/4A protease inhibitors.

### Liver damage induced by NS3/4A protease expression

To investigate potential hepatotoxicity resulting from the expression of NS3/4A protease in the livers of the triple-transgenic mice, liver tissue biopsies were stained with H&E, and serum alanine aminotransferase (ALT) and aspartate aminotransferase (AST) levels were evaluated.

Histological examination showed that hepatic plates and hepatocytes were clear, and the structures of the liver lobule and liver sinusoid were obvious in the three groups. No apparent hepatopathological alterations were observed in liver tissue sections from the *NS3/4A* transgenic mice or triple-transgenic mice compared with the WT mice after 3 days of Dox induction (Fig 4B).

As shown in Fig 4C, the ALT and AST levels in the triple-transgenic mice were not significantly different than those in the *NS3/4A* transgenic mice and the WT mice after 3 days of Dox induction. These results indicated that hepatic expression of NS3/4A protease for 3 days may not induce spontaneous liver damage.

### Telaprevir and boceprevir inhibit HCV NS3/4A protease activity in triple-transgenic mice

To determine whether our created triple-transgenic mouse model is an effective platform for evaluating the effects of NS3/4A protease inhibitors, the abilities of telaprevir and boceprevir to suppress plasma Gluc activity were evaluated.

**Fig 5:**
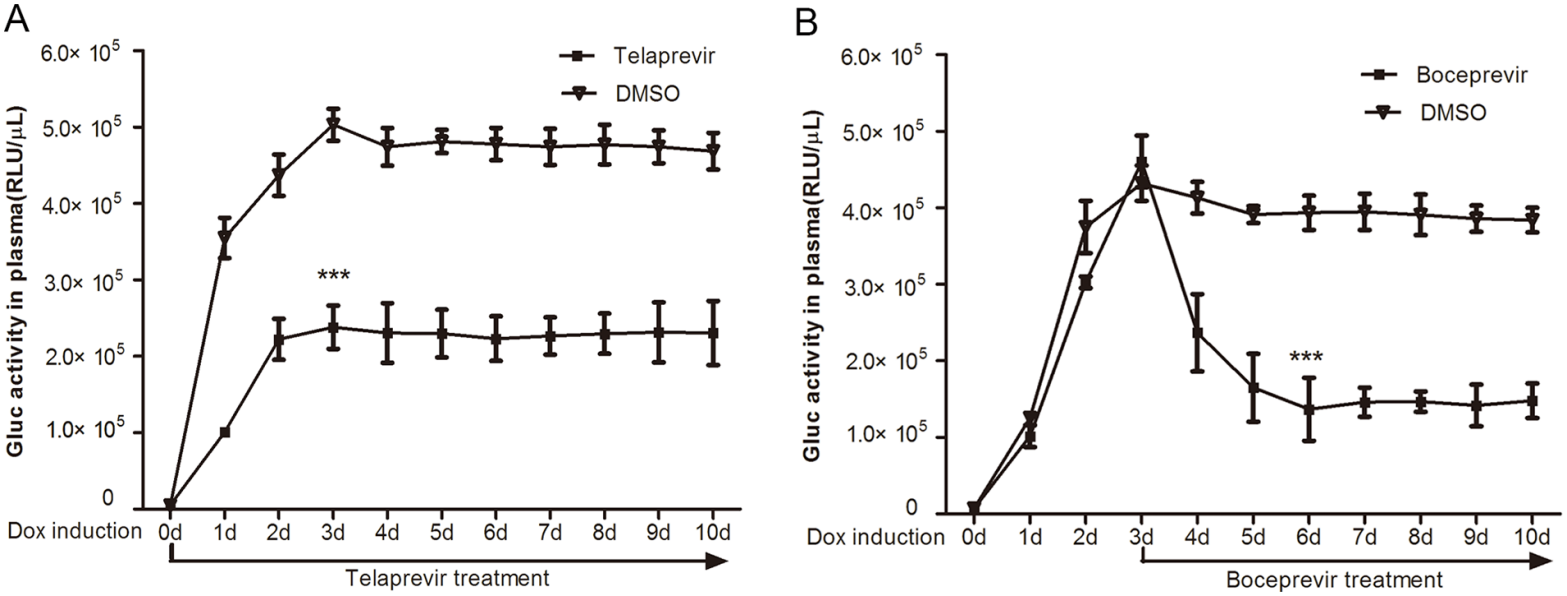
Utilizing the triple-transgenic mouse model to evaluate the effects of NS3/4A protease inhibitors. **(A)** Two groups of triple-transgenic mice (n = 5 per group) were treated with telaprevir (200 mg/kg) or DMSO via oral gavage twice daily for 10 days. During the treatment period, the mice were continuously induced with Dox (1 mg/mL Dox and 50 g/L sugar were dissolved in their drinking water). **(B)** Triple-transgenic mice were induced with Dox for 10 days (n = 5 per group). At the third day after Dox induction, the mice were treated with boceprevir (100 mg/kg) or DMSO via oral gavage twice daily for 7 days. Blood samples were collected daily from the caudal vein to measure plasma Gluc activity. Each data point represents the mean ± SD. *** = p<0.001 compared with the vehicle control.

As shown in Fig 5A, the average plasma Gluc activity in the triple-transgenic mice reached 2.38×10^5^±2.82×10^4^ RLU/μL after 3 days of treatment with telaprevir, whereas the average plasma Gluc activity in control triple-transgenic mice that were treated with DMSO reached 5.03×10^5^±2.09×10^4^ RLU/μL. Thus, treatment with telaprevir reduced Gluc activity by 50% compared with the vehicle control.

Next, triple-transgenic mice were treated with boceprevir (100 mg/kg) after 3 d day of Dox induction, which is when plasma Gluc activity was found to peak (boceprevir group: 4.61×10^5^±3.4×10^4^ RLU/μL plasma, DMSO group: 4.32×10^5^±2.3×10^4^ RLU/μL plasma). The results showed that the average plasma Gluc activity in the triple-transgenic mice decreased to 1.37×10^5^±4.12×10^4^ RLU/μL after 3 days of treatment with boceprevir, whereas the average plasma Gluc activity in the triple-transgenic mice treated with DMSO was 3.94×10^5^±2.23×10^4^ RLU/μL (Fig 5B), corresponding to a 65% reduction in Gluc activity in the boceprevir-treated mice relative to the vehicle control. Notably, Gluc activity gradually reverted back to pre-treatment levels when telaprevir and boceprevir treatments ceased. The results of the above inhibition assays confirmed that our triple-transgenic mouse model is an appropriate platform to evaluate the effects of HCV NS3/4A inhibitors by providing rapid real-time detection of plasma Gluc activity.

## Discussion

In preclinical trials, the HCV NS3/4A serine protease has been explored as a promising target for the development of anti-HCV drugs. However, the lack of a robust small animal model of HCV infection has forced researchers to rely on measurements of anti-HCV activity in cell culture [14–19] and animal pharmacokinetics as surrogate indicators of efficacy prior to human trials [19–21]. Cell-based systems cannot be used to assess the efficacy, toxicity, and bioavailability of new therapeutics *in vivo*, and the majority of currently available mouse models cannot provide rapid real-time assessment of NS3/4A protease inhibitor activity. To overcome these limitations, we previously generated a *NS3/4A* transgenic mouse model [10]. However, in that mouse model, assessment of NS3/4A protease activity was made based on the presence of a SEAP reporter in the blood. This design relied on adenovirus-mediated delivery of the *TM-4B5A-SEAP* reporter gene via injection into the tail vein. Thus, rapid and continuous evaluation of NS3/4A protease inhibitor activity was not possible using this model.

In the current work, we generated a novel triple-transgenic mouse model that expresses the HCV NS3/4A protease in a conditional inducible manner under the control of Tet-On and Cre/loxP systems. This design offers a valuable platform for studying the functions of particular genes *in vivo* [22–25]. Our mouse model contained two different luciferase reporters: firefly luciferase and secreted *Gaussia* luciferase. These luciferase reporters have become powerful tools for BLI-based detection, quantitation and non-invasive monitoring of different biological processes in experimental models. For example, Licui Wang *et al*. used a split firefly luciferase complementation strategy to create a mouse model that enabled noninvasive imaging of NS3/4A serine protease activity [20]. In our triple-transgenic mouse model, the Fluc reporter facilitates rapid screening of genotypically and phenotypically positive transgenic mice via BLI. We observed that approximately 70% of our genotyping-positive triple-transgenic mice exhibited Fluc^+^ expression in the liver. The existence of genotyping-positive Fluc^-^ mice might indicate an insertion of the transgene or invalid transcription, as pronuclear injection can result in random insertion of the transgene into the mouse genome.

We also included the *Gaussia* luciferase reporter gene when creating our triple-transgenic mouse model. This novel secreted reporter from the marine copepod *Gaussia princeps* is 1000-fold more sensitive than SEAP [26,27]. Our results demonstrated that Gluc was successfully secreted into either cell culture medium or the blood of living mice as a result of NS3/4A-mediated excision of the *4B5A* junction. Although we expected to detect a Gluc signal by BLI, the Fluc^+^ triple-transgenic mice submitted to Dox induction did not show a specific bioluminescence signal in the liver area after being injected with coelenterazine. This lack of Gluc signal may have occurred because of Gluc’s susceptibility to tissue absorption and scattering [28]. As expected, Gluc activity in the culture medium of co-transfected cells and in the plasma of triple-transgenic mice submitted to Dox induction was significantly higher than that in *NS3/4A*-transfected cells and *NS3/4A* transgenic mice without Dox induction. The results of cell culture and animal model assays indicated that Gluc can serve as a rapid real-time reporter of NS3/4A protease activity. Furthermore, Gluc activity in the culture medium of *NS3/4A*-transfected cells and in the plasma of *NS3/4A* transgenic mice slightly increased after Dox induction, likely due to endogenous, nonspecific activation of the P_tet_bi-1 promoter. We also observed that Gluc signals in urine samples collected from Dox-induced triple-transgenic mice were markedly higher than those in samples from *NS3/4A* transgenic mice, although the signals were much lower than those measured in plasma samples due to clearance of the reporter through the kidneys (S1 Fig) [29]. Immunohistochemical staining showed that approximately 10% of hepatocytes in Dox-induced triple-transgenic mice expressed NS3/4A protease. This low percentage of NS3/4A-expressing hepatocytes may be due to low copy numbers of the *NS3/4A* transgene, as the transgene was likely randomly inserted into the mouse genome, potentially leading to invalid transcription. In the future, we will further screen our triple-transgenic mouse model using Gluc activity assays to increase the efficiency of detection in the future.

To evaluate whether our triple-transgenic mouse model can serve as an effective platform for evaluating NS3/4A protease inhibitors, mice were treated with telaprevir and boceprevir, and the suppression of plasma Gluc activity was assessed. Telaprevir is a small-molecule peptidomimetic inhibitor of HCV NS3/4A protease with an average binding constant (Ki) of 7 nM [21,30–32]. Perni RB *et al*. reported a ~5-fold reduction in serum SEAP activity in a surrogate mouse model dosed with 25 mg/kg telaprevir [30]. In a 14-day-long phase 1b trial of telaprevir in HCV genotype 1-infected patients, a 4.4 log10 median reduction in plasma viral load was observed in a group of patients dosed with 750 mg of VX-950 every 8 h [32]. Boceprevir is a covalent reversible inhibitor of the HCV NS3 serine protease with an average Ki of 14 nM [33,34]. In the current study, we adopted two methods to evaluate NS3/4A inhibitors. First, triple-transgenic mice were administered telaprevir via oral gavage for 10 days while being continuously induced with Dox. Our data indicated that Gluc activity was reduced by 50% in the telaprevir-treated mice compared with the vehicle-treated mice. Second, triple-transgenic mice were administered boceprevir after full induction of NS3/4A by Dox. The results showed that Gluc activity was reduced by 65% in the boceprevir-treated mice compared with the vehicle-treated mice. Because high concentrations of NS3/4A inhibitors are needed in our mouse model, the model is not appropriate for the analysis of pharmacodynamics, kinetics, or toxicity. However, the model does offer rapid real-time evaluation of HCV NS3/4A inhibitors via the detection of plasma Gluc activity. Additional studies are needed to evaluate the anti-HCV efficiency of other NS3/4A drugs and to identify new NS3/4A inhibitors using our mouse model.

## Conclusions

In the present study, we established a triple-transgenic mouse model that could be conditionally induced to express NS3/4A protease in the liver. This mouse model offers rapid real-time evaluation of NS3/4A protease inhibitors and could serve as an inhibitor screening tool.

## Supporting Information

S1 Fig Analysis of Gluc activity in urine. WT mice, *NS3/4A* transgenic mice and triple-transgenic mice were induced with Dox for 3 days (n = 20 mice/group). Prior to and after Dox induction, 10 μL samples of urine were collected. Luciferase activity was measured over 10 sec using a luminometer (Promega GloMax 20/20). Each symbol represents an individual animal. The horizontal bars indicate group medians. *** = p<0.001 compared with the negative control.
(TIF)
