# Supplementary figures and images for: Conditional Inducible Triple-Transgenic Mouse Model for Rapid Real-Time Detection of HCV NS3/4A Protease Activity
Source: PLoS One. 2016 Mar 4;11(3):e0150894. doi: 10.1371/journal.pone.0150894 (PMC4778798; doi:10.1371/journal.pone.0150894)

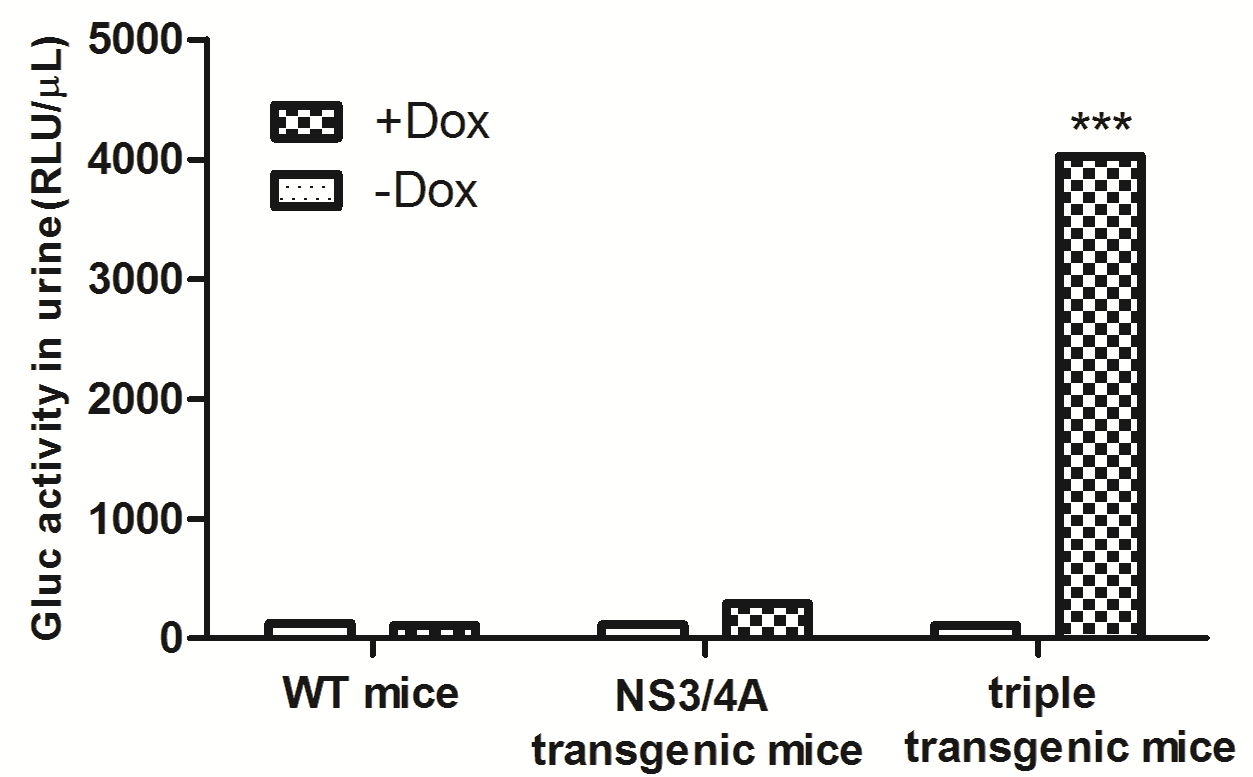

Supplement: S1 Fig — WT mice, NS3/4A transgenic mice and triple-transgenic mice were induced with Dox for 3 days (n = 20 mice/group). Prior to and after Dox induction, 10 μL samples of urine were collected. Luciferase activity was measured over 10 sec using a luminometer (Promega GloMax 20/20). Each symbol represents an individual animal. The horizontal bars indicate group medians. *** = p<0.001 compared with the negative control. (TIF) [file pone.0150894.s001.tif]
